# 肺癌影像学筛查方法的价值比较

**DOI:** 10.3779/j.issn.1009-3419.2010.10.11

**Published:** 2010-10-20

**Affiliations:** 610041 成都，四川大学华西医院呼吸内科 Department of Respiratory Medicine, West China Hospital of Sichuan University, Chengdu 610041, China

**Keywords:** 肺肿瘤, 筛查, 影像学, Lung neoplasms, Screening, Imaging

## Abstract

影像学是肺癌筛查的主要手段，本文综述并比较了常用影像学方法在肺癌筛查中的价值。每种方法各有优缺点，对高危人群采取定期的影像学随访，可以发现更多的早期肺癌，给予及时的治疗。但究竟能否有效降低肺癌的死亡率尚有待于进一步研究。

## Abstract (en)

Radiology is the primary lung cancer screening technique. Whether the mortality rate of lung cancer could be markedly reduce by radiological screening was still unknown to us. It was suggested that high-risk individuals should take regular radiological examinations to detect early lung cancers, followed by suitable treatment. In this review, we compared the values of different radiological methods in lung cancer screening.

肺癌是目前世界上最常见的恶性肿瘤之一，全球每年约有135万人被确诊为肺癌，120万人死于肺癌^[1]^。美国癌症协会(American Cancer Society, ACS)的监测数据显示：2002年-2006年，美国男、女性肺癌发病率分别为86.4/10万、55.5/10万，仅次于前列腺癌和乳腺癌，居第2位；而死亡率则为70.5/10万、40.9/10万，均居首位^[2]^。据此预测，2010年美国新发肺癌22.2万例，占癌症患者总数的15%；死亡肺癌患者15.7万例，占所有癌症死亡病例的28%^[3]^。随着人口老龄化的加剧，以及生活方式和环境等因素的影响，我国肺癌发病率已由20世纪70年代的第4位上升到第1位，目前仍以年均5%的速度递增，是城乡居民的首要死因^[4]^。近年来，虽然化疗、放疗、生物治疗和外科手术等各种治疗方法不断发展，但由于肺癌起病隐匿，大多数患者就医时已到中晚期，总体5年生存率不容乐观，仅为15%左右^[1]^。有学者报道非小细胞肺癌(non-small cell lung cancer, NSCLC)Ⅰa期、Ⅰb期、Ⅱa期、Ⅱb期、Ⅲa期、Ⅲb期、Ⅳ期患者的5年生存率分别为50%、47%、36%、26%、19%、7%和2%^[5]^；而小细胞肺癌(small cell lung cancer, SCLC)局限期为15%，广泛期则降至3%^[6]^。由此可见，改善肺癌患者预后的关键在于进行行之有效的人群筛查，切实提高肺癌的早期诊断水平，对患者进行及时治疗。

一般来说，“筛查”是指通过快捷的检查方法，在高危人群中发现无症状、但患有某种疾病的个体。作为肺癌筛查的常规方法，要求其对早期病例有足够的敏感性，能在个体出现症状之前查出肺癌；对于查出的早期患者给予积极的干预和治疗，能有效降低肺癌病死率；检查方法简便易行且安全可靠，受检者有良好的耐受性，不出现明显的损伤及副作用，费用合理^[7, 8]^。临床上，肺癌的诊断方法包括影像学、血清肿瘤标志物检测、纤维支气管镜、经皮肺穿刺、手术等。血清肿瘤标志物的低敏感性，后三者检查的侵入性和低耐受性等特点，限制了其在肺癌筛查中的应用。实际工作中，肺癌筛查的主要手段是胸部影像学检查。

影像学筛查包括普通胸部X线摄片(chest X-ray radiography, CXR)、数字化X线摄片(digital radiography, DR)、计算机X线断层扫描(computed tomography, CT)、正电子发射计算机断层扫描(positron emission tomography, PET)等，本文就各种检查方法的价值及比较作一综述。

## 1 CXR

CXR简单、经济，是胸部影像学检查的基本方法。肺是含气器官，能在CXR上形成良好的自然对比，清晰显示骨性胸廓、心脏和肺野等。空间分辨率和密度分辨率是评价图像质量的两个主要指标。空间分辨率是指区分两个距离很近的微小物体的能力，以能分辨最小圆孔的直径(cm或mm)的线对(LP/cm、LP/mm)来表示；密度分辨率则是指影像中能显示的最小密度差异，常用两种组织密度差别的百分比来表示^[9]^。CXR的密度分辨率低，且成像后灰阶和对比度固定，不能对感兴趣区进行调节。这导致CXR对 < 10 mm的病灶、与正常解剖结构重叠及周边肺野病灶显示不佳^[10]^，易造成病灶遗漏，从而影响诊断的准确性。关于CXR在肺癌筛查中的价值，美国国家癌症研究所(National Cancer Institute, NCI)早在1960年即开展了大规模的临床试验进行探讨。55 034例40岁以上的男性被随机分配到试验组和对照组，前者每6个月接受一次CXR检查，共3年；后者仅在3年初、末各进行一次CXR检查。结果显示：试验组诊断肺癌132例，其中58例(43.9%)手术切除，3年中因肺癌死亡62例，死亡率为0.7/(1 000人•年)；对照组诊断肺癌96例，28例(29.2%)手术切除，因肺癌死亡59例，死亡率为0.8/(1 000人•年)。对照组肺癌死亡率是试验组的1.03倍，即相对危险度(relative risk, RR)为1.03，95%CI：0.74-1.42，差异无统计学意义。该试验提示虽然CXR能提高肺癌筛出率和早期诊断率，但不能有效降低肺癌死亡率^[11]^。

Manser等^[11]^纳入了4项随机对照试验对CXR肺癌筛查的意义进行*meta*分析。非频繁CXR筛查组38 635例，629例死于肺癌；频繁CXR筛查组42 668例，710例死于肺癌，RR=1.11，95%CI：1.00-1.23，即频繁筛查组肺癌死亡率反而高于非频繁筛查组。

为了改进筛查方式，NCI建立了“早期肺癌协作组” (The Cooperative Early Lung Cancer Group)开展痰脱落细胞学联合CXR筛查的随机对照试验，其中最为著名的是1971年-1976年的梅奥肺计划(Mayo Lung Project)^[12]^。10 933例45岁以上的吸烟(每天至少1包)志愿者被纳入研究。基线筛查中，CXR发现肺癌患者51例，痰脱落细胞发现17例，二者联合检查发现15例。随后，9 211例被随机分入筛查组和对照组。筛查组418例，每4个月接受1次CXR联合痰脱落细胞检查；对照组4 593例，每年接受1次相同检查。随访6年后，筛查组和对照组分别发现肺癌206例和160例，且筛查组中99例属于早期，占48.1%，而对照组仅有51例(31.9%)属于早期。然而，两组的肺癌死亡率相近，分别为3.2/(1 000人•年)和3.0/(1 000人•年)。因此，研究者认为：每4个月1次的CXR联合痰液细胞学检查与每年1次的筛查相比，并不能降低肺癌死亡率。同期在捷克斯洛伐克进行的一项持续3年的研究^[13]^的结果也与之类似。

基于以上研究，学术界达成共识：CXR不是一种理想的肺癌筛查手段。

## 2 DR

DR通过特殊的探测系统，将X线影像信息转换为数字信号，经过计算机处理后得到图像。与CXR相比，DR具有成像速度快，辐射剂量低，图像清晰等特点^[14]^。DR采样速度达64帧/s，曝光后6 s即可成像，可实现动态检查，且辐射剂量仅为CXR的数十分之一。DR空间分辨率为3.6 LP/mm，密度分辨率12 bit-16 bit(密度分辨率在数字成像系统中用图像的位深等级bit表示)，远高于人肉眼的分辨范围。此外，DR采用高千伏摄影(80 kv-120 kv)，对肺内结节病灶穿透性好；图像后处理功能强大，如窗宽窗位调节、病灶放大、反转等，能清晰显示1 mm-2 mm的细小支气管血管影、2 mm-3 mm的孤立非钙化肺结节影及心影后、肺后基底段、膈下肋骨重叠等部位的结节及病灶分叶、毛刺等征象^[15]^，从而降低检查漏诊率，提高肺内病变的鉴别诊断能力。Woodard等^[16]^对49例患者进行胸部DR和CXR检查，发现DR较CXR的图像质量更好，能更多地显示胸部正常解剖结构和肺内病变。Gramer等^[17]^的研究则认为DR对肺部各区域病变的评价与CXR无明显差异，但对纵隔病变的显示，则明显优于CXR。我国学者蒋磊等^[18]^选取90例经CT证实有肺部非钙化结节的受试者作为病例组，并以90例无结节的健康体检者作为对照，均予DR和CXR检查，结果显示：总体结节显示率、隐蔽部位结节显示率及不同大小结节显示率的DR受试者工作特征曲线下面积(area under the receiver operating characteristic curve, AUC)均显著大于CXR，提示DR在肺部结节病灶，尤其是隐蔽部位和微小结节的检查方面优于CXR。Bartjan等^[19]^2006年-2007年对4 938例个体进行胸部影像学筛查，诊断肺癌65例，其中55例患者同时行DR和CT检查；72例对照个体因CT提示肺部非结节性病灶而行DR检查，据此计算DR的AUC为0.52-0.69；对于明显存在的肺部结节，DR提示恶性诊断的敏感度为18%-49%，特异度为92%-100%，而对于可能存在的结节，其敏感度和特异度则分别为36%-73%、82%-99%。可见，DR较CXR是一种更好的检查手段，但其诊断价值仍比较有限。

## 3 CT

CT的横断面成像有效地消除了前后组织重叠的影响，以更直观、清晰的方式显示各部位病灶和正常解剖结构，如胸膜下低密度小结节、肺门、后肋膈角等。由于CT的空间分辨率主要取决于检测器的孔径大小、X线管的焦点尺寸及患者与检测器的相对位置等，而CT的检测器孔径多较X线胶片大，因而一般认为CT的空间分辨率不及CXR和DR，而密度分辨率则较高，可达0.5%。随着影像技术的发展，螺旋CT和多排CT的出现进一步提高了CT的诊断价值。螺旋CT扫描速度快，管球转速达到0.5亚秒级，可在15 s内完成全肺扫描，使在一次屏气情况下完成检查成为可能，避免了受检者因呼吸运动而导致的病灶遗漏。有学者对39例个体分别进行普通CT和螺旋CT扫描，结果显示3例在普通CT上仅发现单个结节，但在螺旋CT上则有多个结节；39例普通CT共发现结节497个，螺旋CT发现705个，平均每例患者结节检出数分别为(18±4.5)个和(12.6±3.2)个，*P*=0.01；从结节大小看，普通CT检出 < 5 mm的结节(12.7±3.7)个，螺旋CT(8.4±2.3)个，*P* < 0.05；而5 mm-10 mm结节则分别为(2.9±0.9)个和(2.4±0.8)个，*P* < 0.05。此外，4例受检者在普通CT上出现呼吸运动伪影，而螺旋CT无1例出现。由此可见，螺旋CT较普通CT有更好的成像能力，更高的结节、尤其是小结节的检出水平^[20]^。然而，随着CT的推广应用，其X线辐射剂量也受到越来越多的人的重视。研究^[21]^表明，常规胸部CT检查的辐射剂量约为CXR的100倍。国际放射防护委员(International Commission on Radiological Protection, ICRP)会认为，每增加1 Sv的X线辐射剂量，恶性肿瘤的发病率就增加5/10万^[21]^。如何在保证图像质量的前提下，将辐射剂量减至最低，使人体所接受的放射损害最小，是推广CT检查的重要基础。1990年，Naidich率先提出了“胸部低剂量螺旋CT” (low-dose computed tomography, LDCT)的概念^[22]^。目前多采用降低管电流(一般为25 mA-50 mA)的方法来降低CT扫描的辐射剂量。CXR的辐射射剂量为0.06 mSv-0.25 mSv，胸部普通CT为3 mSv-27 mSv，LDCT为0.64 mSv-0.8 mSv，低于自然本底水平2.4 mSv，且仅为普通CT的26%，可作为肺癌筛查的常规方法^[21]^。此外，多项研究^[23-25]^表明，LDCT在检测肺部小结节、磨玻璃影等方面与普通CT相似。

日本、美国、加拿大等发达国家早在20世纪90年代开始就利用LDCT进行肺癌筛查。Menezes等^[26]^对3 352例50岁以上、吸烟≥30包^•^年、无肿瘤史的个体进行LDCT筛查，将≥5 mm的非钙化结节或≥8 mm的非结节性病灶定义为“阳性”，随访4年。共65例被诊断为肺癌，其中53例(82%)为Ⅰ期或Ⅱ期，52例(80%)接受手术治疗，LDCT在早期肺癌的诊断中的敏感度和特异度分别为87.7%和99.3%。Seki等^[27]^1993年纳入2 120例肺癌高危个体行LDCT检查，83%为吸烟者。在基线筛查中发现19例肺癌，74%为腺癌，58%的患者为Ⅰ期；随访至2004年，新诊断肺癌57例，63%为腺癌，79%为Ⅰ期。由美国康奈尔大学Weill医学院和纽约Presbyterian医院1993年发起、包括7个国家38个单位参与的国际早期肺癌行动计划(International Early Lung Cancer Action Program, ELCAP)是目前关于LDCT肺癌筛查价值的最大规模研究。2007年，Roberts等^[28]^报道了加拿大的研究结果：从2003年1月到2005年12月，1 000例55岁以上、吸烟≥30包*年的个体自愿接受LDCT检查和随访，随访间隔时间及方法取决于结节大小。3年共诊断肺癌20例，19例NSCLC中14例为腺癌或支气管肺泡癌，4例鳞癌，1例大细胞癌；其中15例为Ⅰ期，14例采用手术治疗。同样，来自意大利Ronchi、Novello及日本Kondo等^[29-31]^的研究也与之基本一致，均提示LDCT在早期肺癌筛查中有重要意义。

Muralikrishna等^[32]^近期的一项系统评价分析了6项随机对照试验，总人数超过14 000例。结果显示：LDCT可以发现更多的肺癌，尤其是Ⅰ期及NSCLC，OR值分别为：4.1、3.9和5.5，95%CI分别为2.4-7.1、2.0-7.4和3.1-9.6。

关于LDCT与CX R的诊断价值比较，美国国家癌症研究所(National Cancer Institute, NCI)的一项研究显示：3 318例重度吸烟者中，1 586例行LDCT筛查，1 550例行CXR检查。LDCT组影像学阳性325例(20.5%)，53例(3.0%)行活检，诊断肺癌30例，肺癌筛出率为1.9%，NSCLC 29例，Ⅰ期占53.3%(16例)；CXR组影像学阳性152例(9.8%)，15例(1.0%)行活检，诊断肺癌7例，Ⅰ期6例。LDCT的肺癌筛出率高于CXR^[33]^。I-ELCAP提供的一组数据显示：LDCT筛查出1 000例吸烟者中有233例(23.3%)肺部有非钙化结节，最终27例被诊断肺癌，23例(85%)为Ⅰ期，26例(96.3%)接受手术切除，远高于Mayo Lung Project的51.1%^[12]^；受检者同时行CXR检查，仅68例(6.8%)显示肺部结节，7例提示恶性，4例为Ⅰ期。即：LDCT的肺癌检出率为2.7%，CXR仅为0.7%，*P* < 0.05；Ⅰ期肺癌检出率LDCT和CXR则分别为2.3%和0.4%，*P* < 0.05。15例LDCT显示结节直径≤10 mm的肺癌患者中，仅2例在CXR上被发现^[34]^。法国为期2年的队列研究^[31]^也表明：LDCT在284例受试者中检出6例肺癌(2.1%)，显著高于CXR的0.4%(1/237)。

迄今尚缺乏大规模临床研究来比较LDCT与DR筛查价值。但可以肯定的是：LDCT在肺癌，尤其是早期肺癌的筛查中有重要作用。尽管如此，目前仍无组织推荐进行基于人群的LDCT普查，其根本原因在于没有充分的证据表明LDCT筛查能有效降低肺癌的死亡率；此外也与LDCT的假阳性率较高，易造成过度诊断有关。目前关于LDCT筛查与肺癌死亡率关系的报道，较权威的是2006年发表在NEJM杂志上的I-ELCAP结果。1993年-2005年，共有31 567例无症状的肺癌高危个体进行了LDCT检查及随访，诊断肺癌484例，412例(85.1%)为Ⅰ期，预计10年生存率为88%，95%CI：84%-91%；302例在诊断后1个月内接受手术治疗，预计10年生存率为92%，95%CI：88%-95%。此研究^[35]^表明LDCT既可提高肺癌的早期诊断率，又可明显降低肺癌死亡率。一项包括1994年-2004年发表的12项研究结果的系统评价，其中2项是随机对照实验，其余10项未设对照组。共25 749例高危个体进行了54 342次LDCT扫描，但3项实验无生存结果(包括2项随机对照实验)，2项ELCAP研究的2年生存率为100%(只随访了2年)，仅1项实验报道LDCT筛查出的肺癌患者5年生存率为76.2%。为了进一步探究LDCT究竟能否有效降低肺癌死亡率，美国NCI从2002年9月开始实施一项国家肺癌筛查试验(National Lung Cancer Screening Trial, NLST)^[36]^，该项目对50 000例高危人群的志愿者进行每年1次的LDCT或CXR检查，直至志愿者因肺癌死亡而终止。此外，荷兰和比利时也开展了一项随机对照肺癌筛查试验(Dutch-Belgian Randomized Lung Cancer Screening Trial, NELSON)^[36]^，他们将受试者分为LDCT筛查组和不进行筛查的对照组，持续10年，至2014年观察LDCT对改善肺癌患者死亡率的影响。这些研究成果必将使我们对LDCT的作用有一个全新的认识。

部分研究者不赞成LDCT筛查的另一个理由是LDCT的假阳性率较高。Wilson等^[37]^对3 642例个体进行LDCT检查，基线筛查时1477例(40.6%)发现有肺部结节，36例(1.0%)行开胸手术、胸腔镜、纵隔镜等检查，但均未发癌；至研究结束时，82例行肺叶切除或胸腔镜检查的个体中，28例为良性病变，占活检者的34.1%。Sone和Reich^[38, 39]^则认为LDCT的过度诊断率可达13%和25%。Muralikrishna等^[32]^的系统评价也显示：LDCT的假阳性率比值比(odd ratio, OR)为3.1，95%CI：2.6-3.7，平均每1 000例可发现235个假阳性结节，3.7例接受不必要的手术活检。

据此，部分学者反对开展人群的肺癌LDCT筛查。他们认为LD CT所检出的肺癌，其生物学行为常为惰性，及时的治疗，包括外科手术切除，并不能明显改变疾病的自然病程和患者预后。他们甚至认为LDCT筛查的费用、假阳性等危害超过了其带来的益处，但是这些观点尚不为公认^[44]^。

尽管LDCT在肺癌筛查中的价值有待于进一步探讨，但一般认为LDCT是目前肺癌筛查方法中最简单、最有效的途径。ACS建议：高危个体，尤其是重度吸烟者或有职业性危险因素者，应在与医师充分交流并理解目前LDCT肺癌筛查现状的基础上，自主选择是否进行LDCT筛查^[40]^。

## 4 PET

PET是一种仅用于临床20余年的最新影像诊断技术，属于发射型计算机断层显像(emission computed tomography, ECT)。ECT通过探测放射性药物在体内病变部位与正常组织之间的浓度分布差异而对病灶进行定性诊断。PET的放射性示踪剂多为由可发生正电子衰变的核素所标记的人体内源性代谢物或其类似物，直接参与各种生化反应过程。因此，PET又被誉为“活体生化显像”。当核素发生衰变时，产生的正电子在人体很短的范围(1 mm-2 mm)内与周围组织中的负电子发生湮灭，形成一对γ光子。这两个γ光子能量相同，均为0.511 Mev，运动方向相反，可用两个位置相对的探测器进行符合探测(coincidence detection)，进而将信号送入显像系统生存PET图像。由此可见，与前述各种方法不同，PET主要通过探测正电子放射性核素在体内的分布而反映局部组织的代谢情况，实现解剖结构及生物学功能的双重显像。目前最常用的示踪剂是^18^F标记的脱氧葡萄糖(^18^F-fluoro-2-deoxy-d-glucose, ^18^F-FDG)。肿瘤细胞代谢活跃，^18^F-FDG作为一种与葡萄糖结构相似的标记物注射进入体内后，在肿瘤细胞内聚集，从而对病灶进行定位和定性诊断。临床上多采用局部组织单位质量所摄取的^18^F-FDG量与全身平均单位质量所摄取的^18^F-FDG的量之比，即标准化摄取值(standardized uptake value, SUV)，来衡量病灶的代谢水平。一般认为肺部结节SUV > 2.5则提示恶性。由于在病变早期，局部组织尚未出现形态结构异常之时，PET即可检测到病灶的代谢异常，因此其敏感度和特异度较既往的检查方法均有明显提高。丁其勇等^[41]^对30例的肺癌患者和30例有肺部良性结节的对照行PET检查，结果显示：PET的敏感度和特异度分别达86.7 %和90.0%。

但是，由于示踪剂不仅浓聚于病变部位，而且在示踪剂参与代谢的相关器官中也有较高的浓聚，因此，PET所显示的解剖结构及病灶与邻近组织的关系常不甚清楚；而CT则有较高的密度分辨率，能清晰显示各部位的结构特征。因此，为了充分利用PET和CT的显像优势，2000年推出了PET和CT的整合设备，即PET/CT。Jeonq等^[42]^回顾性分析了100例个体的LDCT、PET和PET/ CT资料，其中良性病变60例，恶性40例。LDCT、PET和PET/CT的敏感度基本一致，分别为82%、88%和88%；特异度PET/CT最高，为77%，LDCT和PET为66%和71%，差异有统计学意义(*P* < 0.05)；且PET/CT的AUC也较LDCT和PET明显增高(*P* < 0.05)。可见，PET/CT在肺癌诊断中可以提供较LDCT和PET更精确的信息。

然而，由于PET/CT的空间分辨率较低，对 < 7 mm的病灶难以准确评估；且SUV值除了受到病灶本身的代谢水平的影响外，还与病灶大小、部位、受检者呼吸运动、血糖水平等因素有关。因此，一些直径较小，或分化较好、生长缓慢的结节，如支气管肺泡癌，可能出现假阴性的结果；而代谢较活跃的炎性病灶、肉芽肿性结节等，则可能出现假阳性。Kagna等^[43]^观察了93例个体，其中肺癌35例，8例被PET/CT判断为阴性；58例对照中10例被PET/CT判断为阳性，即PET/CT的假阴性率为22.9%，假阳性率为20.8%，不容忽视。此外，PET/CT昂贵的检查费用，也限制了其在肺癌普查中的广泛应用。目前国内外均尚未将PET和PET/CT列为肺癌筛查的常规项目，主要用于对X线检查方法不能确定性质的病变的进一步检查及确诊肺癌患者的临床分期、疗效评估等。

## 5 其他

随着计算机技术的发展，电子计算机辅助诊断(computer aided diagnosis, CAD)作为一种“自动化”诊断方法逐步在临床工作中实施应用。目前已开发出胸DR和LDCT的CAD系统并获得认证。CAD利用专有的信号软件，对图像进行处理和分析，以达到检出微小病灶、鉴别病变性质、提高诊断准确性、缩短工作时间的目的。Sahiner等^[45]^组织6名放射科医师独立阅读85例受检者的胸部LDCT资料，共检出241个直径3.0 mm-18.6 mm的结节。对于3 mm、4 mm、5 mm和6 mm的小结节，6名医师结节检出的准确度为66.1%、72.9%、79.3%和83.8%；结合CAD，其准确度则分别上升至70.5%、76.3%、81.0%和86.2%。其中对于3 mm和4 mm的小结节，两种诊断方法差异有统计学意义，*P*分别为0.002、0.020。此外，对于3 mm的小结节，医师诊断的敏感度为56%，结合CAD则提高到67%。Das等^[46]^的研究也证实CAD可有效地提高肺部结节、特别是小结节的检出率和准确度。但是，CAD并不是一种完美的诊断手段，居高不下的假阳性率就是突出的问题之一。Roos等^[47]^的研究显示医师读片时平均每个病例出现1.15个假阳性结节；联合CAD诊断则平均每个病例出现1.45个假阳性结节。此外，CAD的结果还易受到扫描剂量、患者呼吸运动、身体扭动、心脏搏动等因素的影响。因此，临床医师应认识到：CAD并非万能的“自动化”诊断方法，也有其局限性，诊断结果首先取决于医师自己的读片水平。

磁共振成像(magnetic resonance imaging, MRI)虽然是一种先进的显像方法，但其成像的原理及较低的空间分辨率决定了它对肺部病灶的显示不够清晰，一般不用于肺部结节的筛查。然而，MRI的密度分辨率较高，且对血管显示较清楚，因此可用于甄别肺门或纵膈肿块与邻近血管的关系。近年来开展的MRI功能成像尚在逐步完善之中，或许能为今后的肺癌筛查提供新的手段。

纤维支气管镜、支气管腔内超声(endobronchial ultrasound, EBUS)等检查可以直视部分病灶，并可获得病理活检组织，但其侵入性的特点使之不能作为肺癌普查的方法。

综上所述，目前尚无一种完全理想的肺癌影像学筛查方法，各种检查手段均存在一定的缺陷。学术界关于是否应该推广肺癌筛查亦存争议。尽管如此，基于已有的研究成果，部分学者仍建议对肺癌高危人群进行定期的、以LDCT为首选方法的肺癌筛查，以期发现更多的早期肺癌并给予及时的治疗。
